# Potential Role of Inflammasomes in Aging

**DOI:** 10.3390/ijms26146768

**Published:** 2025-07-15

**Authors:** Gilyoung Lee, Geun-Shik Lee

**Affiliations:** College of Veterinary Medicine and Institute of Veterinary Science, Kangwon National University, Chuncheon 24341, Republic of Korea

**Keywords:** inflammaging, aging, immunosenescence, NLRP3, inflammasome

## Abstract

The aging process is associated with the emergence of low-grade, sterile inflammation, called inflammaging, which can accelerate aging-related diseases, such as neurodegenerative, cardiovascular, and musculoskeletal diseases. Recent studies have focused on the novel concept that inflammasomes represent a key innate immune pathway, mechanistically participating in aging-induced stress recognition. This review summarizes the advancements in inflammasome research related to aging. Particular attention is given to the close relationship between aging and inflammasomes and how these processes impact the health of the elderly. Inflammaging has various causes, such as metabolic disorders, changes in the gut microbiota, and immunosenescence. Hence, the connection between inflammasomes and these causes must be explored. This paper describes inflammasomes as a significant contributing factor among the mechanisms that make individuals susceptible to aging-related diseases and discusses the potential role of inflammasome regulation in effectively counteracting aging.

## 1. Introduction

The aging population is growing at an unprecedented rate, particularly in developing countries [1]. In 2019, the population aged 60 years and above was 1 billion and has been projected to reach 1.4 billion and 2.1 billion by 2030 and 2050, respectively. As human life expectancy increases, the prevalence of diseases, such as neurodegenerative diseases, cardiovascular diseases, diabetes, and cancer, is becoming a significant public health concern [1,2]. Kuan et al. [3] proposed the term “aging-related” to describe diseases that become more prevalent with advancing age, such as those mentioned in the previous sentence, suggesting a stronger mechanistic connection to the aging process itself [4]. Aging is represented by the accumulation of various changes generated in cells and tissues over time [5]. This process leads to cellular dysfunction and diminished tissue regeneration, making it a powerful risk factor for various chronic inflammatory conditions [2]. The demographic shift toward an older population poses a significant socio-economic burden that society and healthcare systems must manage in the coming decades [6].

Inflammaging is a term used to describe the physiological changes in the immune system associated with aging that play a significant role in the onset and progression of complex aging-related diseases [7]. These changes affect various conditions, including skin aging, cardiovascular disease, neurodegenerative disease, periodontal disease, and other chronic illnesses [8,9,10,11]. Molecular and cellular mechanisms linking aging and chronic inflammation have been studied extensively, focusing on increased cytokine expression related to inflammasomes and their sustained activation in inflammatory diseases [2,12,13]. This review summarizes new research findings related to inflammasomes as common therapeutic targets for inflammatory conditions and diseases in aging.

## 2. Molecular Mechanisms of Inflammasome Activation

Inflammasomes are protein complexes observed within the cell cytoplasm, serving as critical molecular platforms that induce inflammatory responses [14]. Inflammasomes recognize pathogen-associated molecular patterns (PAMPs) and damage-associated molecular patterns (DAMPs), leading to the secretion of pro-inflammatory cytokines, such as interleukin (IL)-1β and IL-18, as well as the initiation of pyroptosis, a form of cell death [15]. The activation of inflammasomes involves various sensors, including nucleotide-binding oligomerization domain and leucine-rich repeat-containing receptor (NLR) proteins, pyrin, absent in melanoma 2 (AIM2), and gamma-interferon-inducible protein Ifi-16 (IFI16) [14]. These sensors activate the protease enzyme caspase-1, which cleaves pro-IL-1β and pro-IL-18, generating mature IL-1β and IL-18. Furthermore, inflammasome activation leads to the cleavage of gasdermin-D, resulting in N-terminal fragments that form pores in the cell membrane. This process induces pyroptosis while releasing various DAMPs and cytokines. Inflammasomes play a fundamental role in enhancing innate immune responses and promoting pathogen clearance and tissue repair. However, their activation can be context-dependent, and excessive activation may exacerbate inflammatory conditions. Conversely, insufficient cytokine activation could contribute to chronic inflammation. Therefore, the precise regulation of inflammasome activity is essential for maintaining physiological homeostasis.

Inflammasomes play a key role in innate immunity and inflammation. Their activation is tightly regulated and occurs in response to various danger signals, including PAMPs and DAMPs [16]. Emerging evidence suggests that several inflammasomes also play pivotal roles in aging-related diseases [2,17,18]. These studies highlight the significance of innate immunity during the aging process, emphasizing the role of inflammasomes in aging-related changes (Table 1). Aging-related changes and inflammasome dysregulation involve similar molecular mechanisms [19]. When inflammasomes are not adequately regulated, they show similar characteristics to those seen in aging [19]. Furthermore, exploring the potential of targeting inflammasomes to modulate inflammaging and promote healthy aging [20] is an area of active investigation.

## 3. Role of Inflammasomes in Immunosenescence

In recent years, research on the intricate relationship between inflammation and aging has been increasing steadily, particularly how inflammation exacerbates aging and causes aging-related diseases [30,31]. Aging is an inevitable process for most organisms. On the other hand, it is accompanied by gradual functional decline and increased susceptibility to various diseases, including neurodegenerative, cardiovascular, and musculoskeletal diseases [30].

### 3.1. Adaptive Immunity

Thymic involution is one of the most dramatically recognized aging-related changes in vertebrates [32]. The characteristics of aging-related thymic involution include a decrease in tissue mass, the loss of tissue organization, and abnormal structure, ultimately leading to a reduced output of naive T cells [32]. As aging progresses, the differentiation capacity of T cells declines, leading to inefficient immune responses. Consequently, immunity weakens, reducing the effectiveness of immunization, increasing susceptibility to infections [33], and eliminating infected, damaged, or cancerous cells [34]. Furthermore, T cells regulate the function of macrophages and dendritic cells, ensuring that these innate immune cells effectively recognize pathogens and present antigens. These age-related immune alterations contribute to increased infection rates, diminished vaccine efficacy, and weakened tumor immune surveillance, further accelerating inflammaging, a state of chronic low-grade inflammation associated with aging. In mouse models, increased NLRP3 inflammasome activation has been detected in the thymus with aging [23]. The age-related accumulation of lipotoxic danger signals, such as free cholesterol and ceramides, was shown to induce NLRP3-dependent caspase-1 activation in the thymus, leading to thymic involution. The deletion of NLRP3 and ASC lowered caspase-1 activation and increased thymopoiesis, resulting in reduced age-related thymic atrophy, the preservation of cortical thymic epithelial cells, and the maintenance of T cell repertoire diversity [23]. These results indicate that inflammasome-driven metabolic stress contributes to age-associated thymic degeneration and immunosenescence.

Similarly, B cells play a critical role in immune regulation beyond their function in antibody production. Various B cell subsets act as immune regulators and antigen-presenting cells, facilitating T cell activation and modulating immune responses. However, aging leads to a decline in B cell numbers, alterations in B cell composition, and diminished antibody responses, which significantly influence the onset and progression of infections and multiple autoimmune diseases [35]. Lipolysis declines progressively with age [36]. This impaired mobilization of free fatty acids in older individuals is associated with failure to maintain homeostasis and decreased survival under fasting conditions [36]. A separate study demonstrated that resident non-senescent aged adipose B cells express IL-1R, proliferate in response to IL-1 signaling, and suppress lipolysis during aging [37]. Thus, impaired adipose tissue function in aging is closely linked to B cell homeostasis, and the NLRP3 inflammasome has emerged as a key regulator of immunosenescence, age-related inflammation, and metabolic dysfunction [2,38,39,40].

Given these points, immunosenescence in adaptive immune responses involving T and B lymphocytes help increase susceptibility to infections and chronic inflammation in older individuals. T and B cells are not merely immune cells but serve as “commanders” and “archivists” that regulate immune responses, by activating B cells, promoting antibody production, and modulating innate immunity. Their characteristics are rooted in their memory and orchestration abilities.

### 3.2. Innate Immunity

In addition to T and B cells, innate immune cells, particularly macrophages, also exhibit memory-like properties, playing a crucial role in immunosenescence and inflammaging. While traditionally considered part of the immediate immune response, macrophages possess a form of innate immune memory known as “trained immunity,” allowing them to modify their response based on past encounters with pathogens or inflammatory stimuli [41,42]. However, with aging, macrophage function becomes dysregulated, leading to impaired phagocytosis, altered cytokine production, and a persistent pro-inflammatory state. This shift contributes to chronic, low-grade inflammation, or inflammaging, which exacerbates tissue damage and increases susceptibility to infections and age-related diseases [43]. The age-related decline in macrophage plasticity not only weakens their ability to clear pathogens and apoptotic cells but also fuels systemic inflammation, further disrupting immune homeostasis [44]. Consequently, macrophages emerge as key players in the aging immune system, bridging the innate and adaptive responses while shaping the inflammatory landscape that underlies immunosenescence [45]. Overall, aging leads to a decline in the innate and adaptive immune functions, with macrophages playing a crucial role in this process. Impaired macrophage activity helps reduce phagocytosis, decreases antigen presentation, and dysregulates inflammatory responses, ultimately compromising immune resilience and tissue homeostasis.

### 3.3. Inflammaging

In contrast to the overall decline in immune function observed during aging, the body exhibits chronic, low-grade, sterile inflammation [7]. This persistent inflammatory state, often referred to as “inflammaging”, is frequently cited as a significant factor contributing to the increased prevalence of aging-related diseases [46,47]. Unlike acute inflammation triggered by infections or injuries, inflammaging persists over time and is associated with various aging-related pathological conditions, which are believed to result from the dysregulation of innate immunity because of chronic exposure to metabolic stress and endogenous danger signals, such as high glucose, adenosine triphosphate (ATP), cholesterol, and uric acid [48]. They can activate inflammasomes, intracellular protein complexes that regulate inflammation and cell death [14,16]. Among inflammasomes, the NLRP3 inflammasome is particularly relevant to inflammaging due to its role as a sensor for a wide range of metabolic signals [49]. In fact, NLRP3 has a critical role in arteriosclerosis and chronic inflammation [25], and in addition, the NLRP3 inflammasome acts as a major mechanism in many age-related diseases [19]. Moreover, recent findings indicate that other inflammasomes, previously thought to be activated primarily by specific PAMPs, can also be triggered by aging-related metabolic stressors and dysfunction [17,29]. As a result, the role of inflammasomes in inflammaging has become increasingly diverse, prompting further research into their significance in age-related inflammation and metabolic disorders. Further research is needed to elucidate the precise mechanisms by which NLRP3 and other inflammasomes contribute to inflammaging and whether targeting these pathways could offer therapeutic benefits in age-related inflammatory disorders. Details on aging-related diseases will be addressed in more detail in the following chapter on therapeutic strategies.

## 4. Therapeutic Strategies and Inhibitors

**Figure 1:**
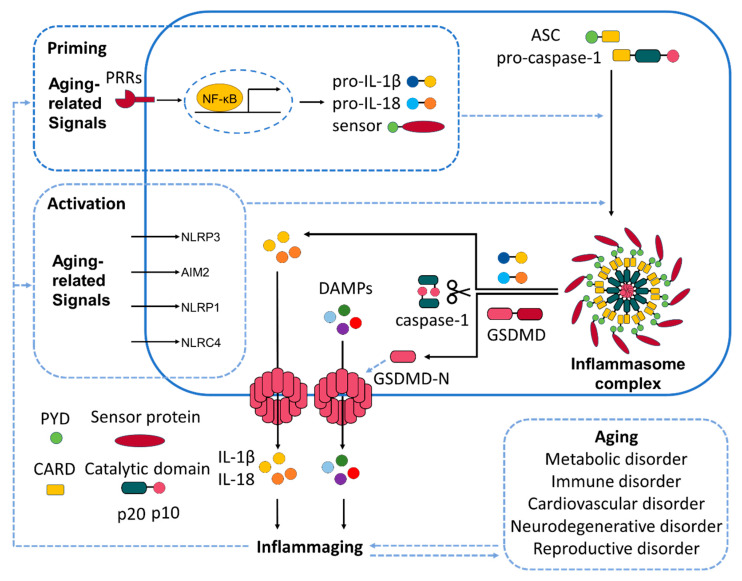
Inflammasome activation pathway and aging. Molecular pathways of inflammasome activation which are critically involved in inflammaging and aging. Each inflammasome sensor responds to age-associated DAMPs. Upon activation, these inflammasome complexes recruit adaptor protein ASC and activate caspase-1. This leads to cleavage of pro-inflammatory cytokines IL-1β and IL-18 into their active forms and promotes gasdermin D-mediated membrane pore formation, resulting in pyroptotic cell death. Release of these cytokines and pyroptotic debris amplifies systemic inflammation and contributes to chronic low-grade inflammation observed in aging and age-related diseases. Black solid arrows indicate mechanisms that have been clearly elucidated, whereas blue dashed arrows denote mechanisms for which conclusive evidence has not yet been established.

NLRP3, NLRP1, and NLRC4, along with AIM2 and the pyrin inflammasome, have been implicated in age-related inflammation and chronic diseases [50,51]. Inflammasome activation is linked to metabolic disorders, cardiovascular diseases, neurodegeneration [14], and inflammaging [2], making them attractive targets for anti-inflammaging interventions (Figure 1). Among various aging-related diseases, the roles and pathological features of individual inflammasomes were described in neurodegeneration (Table 2).

### 4.1. Key Inflammasomes Driving Inflammaging

#### 4.1.1. NLRP3

The NLRP3 inflammasome is considered a central driver of inflammaging. NLRP3 is broadly expressed in immune and parenchymal cells and is activated by a wide array of age-related danger signals (e.g., extracellular ATP, cholesterol crystals, β-amyloid, urate, and other DAMPs) that accumulate with aging [58]. NLRP3 activation requires a two-step process: a priming step (often via NF-κB signaling from Toll-like receptors) that upregulates NLRP3 and pro-IL-1β and a trigger step (such as mitochondrial dysfunction, lysosomal rupture, or ionic flux) that causes the NLRP3 sensor to assemble with the ASC adaptor and caspase-1 [59]. This leads to the release of IL-1β and IL-18, potent inflammatory cytokines. Mechanistically, chronic NLRP3 activation is believed to sustain the “smoldering” inflammation of aging. For instance, NLRP3 inflammasome activity is elevated in aged tissues and promotes tissue damage [60]. A well-documented example illustrating the link between inflammasomes and inflammaging is AD. In AD, β-amyloid (Aβ) deposits are detected as DAMPs by microglial TLRs, which enhance the priming phase of inflammasome activation. Subsequent stimuli such as extracellular ATP, ROS, and ionic flux further trigger the assembly of the NLRP3 inflammasome complex, leading to caspase-1 activation and the release of pro-inflammatory cytokines IL-1β and IL-18. This sustained cytokine production maintains chronic neuroinflammation within the brain. Notably, IL-1β has been shown to promote tau hyperphosphorylation and the formation of neurofibrillary tangles, ultimately exacerbating neuronal damage and cognitive decline [61,62]. In mice, the inhibition or genetic deletion of NLRP3 mitigates many aging phenotypes, improving metabolic health, reducing thymic atrophy, preserving bone density, and even enhancing female reproductive longevity [23,63,64]. Age-related shifts in the gut microbiota can also be sensed by the NLRP3 inflammasome, promoting intestinal and systemic low-grade inflammation that accelerates inflammaging processes [22]. Notably, NLRP3-deficient mice are protected from age-related inflammation. They show an attenuation of degenerative changes and a ~30% extension in lifespan compared to wild-type mice [60]. These findings establish NLRP3 as a key instigator of inflammaging and a prime target for intervention.

#### 4.1.2. NLRP1

NLRP1 is highly expressed in barrier tissues and the brain. While its activators include viral RNA and bacterial toxins [65], evidence suggests that NLRP1 contributes to sterile inflammaging, particularly in the brain. The protein levels of NLRP1 and caspase-1 increase in the aging mouse brain (e.g., hippocampus) [18]. Activated NLRP1 in neurons can drive neuroinflammation and has been implicated in cognitive decline and neurodegenerative diseases [18]. In vivo, NLRP1 has been linked to age-related neurobehavioral changes; for example, it may contribute to learning impairments under chronic stress in older animals [18]. Inhibiting NLRP1 (e.g., probenecid) was shown to reduce neuronal senescence, damage markers, and spatial learning deficits in aged mice [18]. Unlike NLRP3, no specific NLRP1 inhibitor is clinically available, but these insights highlight NLRP1 as another contributor to inflammaging, especially in the context of brain aging and the senescence–inflammation feedback loop.

#### 4.1.3. AIM2

The AIM2 inflammasome is a cytosolic sensor for cytosolic DNA and is increasingly recognized as a contributor to inflammaging. While AIM2 plays a key role in host defense, its chronic activation in aging tissues can exacerbate inflammation and tissue damage. One notable context in which AIM2 contributes to inflammaging is clonal hematopoiesis of indeterminate potential (CHIP), a condition where hematopoietic stem cells (HSCs) acquire mutations that drive low-grade systemic inflammation. CHIP prevalence increases with age, affecting over 10% of individuals older than 70 years [25]. Importantly, CHIP carriers have a heightened risk of developing atherosclerosis, a localized vascular disease in which arterial plaque builds up and becomes inflamed [25]. Recent findings highlight AIM2 as a mechanistic link between age-associated CHIP and atherosclerosis progression. In a murine model of CHIP (JAK2V617F mutation, commonly found in elderly individuals with CHIP), AIM2 activation in myeloid cells was shown to drive excessive IL-1β release, macrophage proliferation, and plaque destabilization, all of which accelerate atherosclerosis. Mice deficient in AIM2 exhibited smaller and more stable atherosclerotic plaques despite carrying the JAK2 mutation, indicating that AIM2 contributes to the localized vascular inflammation seen in atherosclerosis while being systemically fueled by the aging-associated CHIP environment [25]. Given these findings, therapeutic interventions aimed at blocking AIM2-driven inflammation could help interrupt this age-related cardiovascular inflammation.

#### 4.1.4. NAIP/NLRC4 Inflammasomes

The NAIP/NLRC4 inflammasome is traditionally associated with bacterial infections [66], but emerging evidence suggests that it also plays a role in inflammaging. NLRC4 activation triggers caspase-1-mediated IL-1β and IL-18 release, promoting immune activation and pyroptotic cell death [66]. A key study on NAIP/NLRC4 and inflammaging found that aged tissues show increased NLRC4 inflammasome activity, characterized by elevated expression levels of IL-1β, IL-18, ASC, and caspase-1 in the brains and immune cells of aged mice [67]. NAIP/NLRC4 expression was significantly elevated in hippocampal and cortical lysates from aged animals, correlating with increased levels of oxidative stress markers and pro-inflammatory cytokines [67]. In addition to its role in the brain and immune system, NLRC4 contributes to vascular and metabolic aging. In aged mice, NLRC4 activation was linked to increased arterial stiffness and elevated blood pressure, partially driven by IL-1β–mediated endothelial dysfunction [67]. Furthermore, altered nucleotide metabolism in elderly individuals was found to prime and activate the NLRC4 inflammasome, exacerbating age-related inflammation [17]. Further supporting its relevance in aging, a large-scale transcriptomic study demonstrated that elderly individuals could be categorized into distinct health states based on their inflammasome gene expression profiles [17]. Those with high NLRC4 expression exhibited increased systemic inflammation, hypertension, and metabolic dysregulation, whereas those with lower expression had a healthier immune profile and improved metabolic markers [17]. This suggests that NLRC4 may be a marker of inflammaging severity and a potential therapeutic target.

### 4.2. Pharmacological Inhibition of Inflammasomes

Inflammasome inhibition is a promising strategy for reducing inflammaging, but inflammasomes are essential for early immune responses to fight infections. Complete or prolonged inhibition can impair host defense, increasing infection risk [15]. Therefore, inflammasome-targeting therapies must be flexible and context-specific, preventing chronic inflammation while preserving acute immune responses.

In addition, recent studies have highlighted the importance of developing inhibitors that specifically target senescent cells, as cellular senescence and inflammasome activation are closely interconnected. For example, the inflammasome can orchestrate a complex secretory program that drives paracrine senescence [68]. Targeting this interaction may help prevent the propagation of senescent phenotypes and chronic inflammation in aging tissues [69].

Emerging evidence indicates that the pharmacological blockade of the NLRP3 inflammasome/IL-1β loop can mitigate endothelial cell senescence and dysfunction [69], while tissue-specific differences in inflammasome-mediated inflammatory responses further support the need for tailored senescence-targeting approaches [70]. Furthermore, inflammasome-mediated premature immunosenescence has been linked to vascular aging via perivascular adipose tissue dysfunction [71]. Collectively, these findings underscore the potential benefits of developing senolytic or senomorphic strategies that include selective inflammasome inhibition.

#### 4.2.1. Limitations of Current Inhibitors

Existing inhibitors, including anakinra, rilonacept, and canakinumab, mainly block IL-1β signaling but do not directly suppress upstream inflammasome activation or the broader secretory cascades that sustain inflammaging. This means that residual inflammasome priming, pyroptosis, and senescence-associated secretory phenotypes (SASPs) can continue unchecked, fueling local and systemic chronic inflammation [68]. Furthermore, these agents generally suffer from poor blood–brain barrier penetration, limiting their effectiveness in treating neuroinflammatory or neurodegenerative components of inflammaging. In addition, current approaches rarely address tissue-specific variation in inflammasome-driven inflammatory responses [70,72], which suggests that a one-size-fits-all IL-1 blockade may overlook key senescent niches. Although caspase-1 inhibition or IL-18-neutralizing therapies are under investigation as alternative strategies, their long-term selectivity and safety remain to be clarified. In this context, selective inflammasome inhibitors like MCC950 are notable for directly targeting NLRP3. However, because NLRP3 plays important roles in early host defense, uncontrolled or prolonged suppression could weaken resistance to infection or impair beneficial acute immune reactions. Thus, new therapeutic strategies should aim to combine selective inflammasome inhibition with senolytic or senomorphic actions, minimizing chronic inflammation and paracrine senescence propagation while sparing protective immune functions [69,71].

#### 4.2.2. Upstream Inflammasome Inhibition and Anti-Aging Effects

Rather than targeting cytokines only at the downstream level, the direct suppression of inflammasome priming and assembly may offer broader and more durable anti-inflammaging benefits [60]. By interrupting upstream activation, these approaches can simultaneously limit IL-1β and IL-18 secretion, pyroptosis, and the SASP-driven spread of senescence [68].

Recent evidence also supports combining pharmacological NLRP3 blockade with lifestyle or metabolic interventions, such as caloric restriction and exercise, which naturally reduce oxidative stress, improve mitochondrial resilience, and dampen inflammasome activation [54]. Furthermore, because inflammasome activation shows tissue-specific patterns [70] and can contribute to immunosenescence through mechanisms like perivascular adipose tissue dysfunction [71], precision strategies that integrate senolytic or senomorphic compounds could amplify therapeutic effects. By addressing the inflammasome’s dual role in both innate immunity and cellular senescence, upstream modulation holds promise for delaying multiple age-related pathologies, including vascular aging, endothelial dysfunction, and neuroinflammation [69].

#### 4.2.3. Alternative NLRP3 Inhibitors: Sulfur-Containing Compounds

Sulfur-containing compounds have emerged as promising NLRP3 inflammasome inhibitors, with some demonstrating anti-inflammatory effects through distinct molecular mechanisms. These compounds may offer therapeutic potential for inflammaging by modulating oxidative stress, inflammasome priming, and cytokine release. Disulfiram, an FDA-approved drug for alcoholism, has been found to block NLRP3 inflammasome activation by blocking gasdermin D pore formation [73]. This results in reduced pyroptosis and cytokine release, making disulfiram a strong candidate for repurposing in chronic inflammatory conditions [73]. Another sulfur-based compound, dimethyl sulfoxide (DMSO), has been identified as an inhibitor of ROS-dependent NLRP3 activation. Since mitochondrial reactive oxygen species (ROS) are a known trigger for NLRP3 inflammasome priming and activation, DMSO may exert anti-inflammatory effects by mitigating oxidative stress and reducing NLRP3-dependent IL-1β release [74]. Similarly, methylsulfonylmethane (MSM) has shown the ability to suppress NLRP3 transcription and inhibit inflammasome-driven cytokine release. Studies suggest that MSM interferes with NF-κB signaling, inflammasome priming, and mitochondrial dysfunction, all of which are key contributors to age-related chronic inflammation [75]. The broad anti-inflammatory profile of MSM, combined with its well-established safety as a dietary supplement, supports further investigation into its role in targeting inflammaging [75].

#### 4.2.4. Natural Compounds and Nutraceuticals for Inflammasome Modulation

Several food-derived and botanical compounds have demonstrated inflammasome inhibitory effects, often through pleiotropic mechanisms, including NF-κB suppression, mitochondrial protection, and the direct inhibition of inflammasome components. Given their long-term safety profiles, these compounds offer an attractive approach for mitigating inflammaging and age-associated diseases. Caffeine has been shown to inhibit NLRC4-driven IL-1β production, providing potential anti-inflammatory benefits in aging. A study on older individuals demonstrated that caffeine inhibited metabolite-induced IL-1β release, suggesting that its consumption may contribute to reduced systemic inflammation and improved metabolic health in aging populations [17]. Korean Red Ginseng (Panax ginseng) has also exhibited inflammasome-suppressing effects, particularly in inhibiting both NLRP3 and AIM2 inflammasome activation. Ginsenosides, the active components in ginseng, reduce IL-1β and IL-18 secretion, likely by interfering with inflammasome priming, NF-κB activation, and oxidative stress pathways. These properties make Korean Red Ginseng a promising natural anti-inflammaging intervention, with potential benefits in cardiovascular, metabolic, and neurodegenerative diseases [76]. Similarly, blueberry polyphenols, such as quercetin and anthocyanins, have been reported to inhibit NF-κB activation and inflammasome signaling, thereby reducing inflammatory cytokine production [77,78]. These polyphenols may enhance mitochondrial function, suppress ROS generation, and limit inflammasome priming, making blueberries a valuable dietary component in controlling age-related inflammation and metabolic dysfunction [78]. Ashwagandha (Withaferin A) is another natural compound that exhibits dual inflammasome-suppressing properties. It reduces NF-κB activity and caspase-1 activation, leading to decreased pyroptotic cell death and inflammatory cytokine release. These effects suggest that ashwagandha may help counteract chronic inflammation in aging-related diseases such as neurodegeneration, cardiovascular disease, and metabolic disorders [79]. Curcumin, a polyphenol from turmeric, exhibits complex effects on inflammasomes. It inhibits NLRP3 inflammasome activation by preventing K+ efflux and disturbing downstream events, including the efficient spatial arrangement of mitochondria, ASC oligomerization, and speckle formation [80]. Conversely, in acute myeloid leukemia (AML) cell lines, curcumin has been found to activate the NLRC4, AIM2, and IFI16 inflammasomes, inducing pyroptosis through the upregulation of the ISG3 transcription factor. These dual actions suggest that curcumin’s impact on inflammasomes is context-dependent, necessitating a nuanced approach in anti-inflammaging strategies to balance its inhibitory and activating effects [81]. Lastly, riboflavin (Vitamin B_2_) has demonstrated the ability to reduce mitochondrial ROS production, thereby inhibiting NLRP3, NLRC4, and AIM2 inflammasome activation. Given its crucial role in mitochondrial metabolism and redox balance, riboflavin supplementation may serve as a simple yet effective strategy to mitigate inflammasome-driven inflammation in aging individuals [82].

## 5. Conclusions and Future Perspectives

The targeting of inflammasomes offers a promising avenue for mitigating inflammaging and age-related diseases. Given the distinct yet overlapping roles of various inflammasome sensors, the development of selective and broad-spectrum inflammasome inhibitors is critical. While many studies have focused on NLRP3 inhibition, the involvement of other inflammasomes in inflammaging suggests that a more comprehensive approach is necessary. Each inflammasome sensor responds to different activation signals, meaning that a single-target strategy may be insufficient to fully mitigate chronic inflammation and its systemic effects in aging. Further research is needed to determine how targeting multiple inflammasomes simultaneously could impact the inflammaging process and whether dual or multi-inflammasome inhibition can provide synergistic benefits without compromising immune surveillance. Sulfur-containing compounds (e.g., disulfiram, DMSO, MSM) and food-derived inhibitors (e.g., caffeine, Korean Red Ginseng, blueberry polyphenols, curcumin, ashwagandha, and riboflavin) have demonstrated potential in modulating pyroptosis and reducing inflammatory cytokine production. However, the challenges of bioavailability and targeted delivery must be addressed to maximize therapeutic efficacy. Future studies should focus on enhanced formulations and combination therapies to optimize anti-inflammaging interventions while maintaining immune homeostasis. These strategies will be critical in developing clinically viable inflammasome-targeting therapies that promote healthy aging and longevity.

## Figures and Tables

**Table 1:** Aging-related danger signals.

Aging-Related Signal	Description	Detected by	References
Gut microbiome change	Dysbiosis and altered microbial metabolites influencing inflammation.	NLRP3 inflammasome	[21,22]
Free cholesterol, ceramides	Lipid molecules that accumulate in aging tissues, contribute to inflammation.	NLRP3 inflammasome	[23]
ROS	Highly reactive molecules are formed by the incomplete reduction of oxygen during cellular respiration.	NLRP3, AIM2 inflammasome	[24,25]
MSU (monosodium urate)	Crystals formed due to hyperuricemia, which is associated with gout.	NLRP3 inflammasome	[13]
Uric acid	Elevated levels lead to crystal formation associated with gout.	NLRP3 inflammasome	[26,27]
Cholesterol crystals	Formed in atherosclerosis, they contribute to plaque formation.	NLRP3 inflammasome	[19]
Oxidized LDL	Modified form of low-density lipoprotein, contributes to atherosclerosis.	NLRP3 inflammasome	[19]
α-synuclein	Aggregates in Parkinson’s disease, forms Lewy bodies.	NLRP3 inflammasome	[28]
Adenine and N4-acetylcytidine	Nucleotide-derived metabolites.	NLRC4 inflammasome	[17]
dsDNA	DNA fragments released into the cytoplasm due to cellular damage or stress.	AIM2 and IFI16	[29]
Unknown agent	Associated with cognitive impairment in the hippocampus.	NLRP1	[18]

**Table 2:** Inflammasome in neurodegeneration.

Inflammasome	Key Cells	Trigger	Pathology	References
NLRP1	Cortical and hippocampal neurons	Amyloid-β (Aβ) aggregates activate neuronal NLRP1 directly	Caspase-1 activation in neurons → IL-1β, IL-18 maturation → neuronal pyroptosis → cognitive impairment and neurodegeneration	[52,53]
NLRP3	Microglia	Aβ plaques and tau tangles induce microglial phagocytosis → lysosomal rupture → cathepsin B release → NLRP3 activation; also primed by TLR/NF-κB signaling	Sustained IL-1β and IL-18 release → chronic neuroinflammation, impaired Aβ clearance, microglial M1 polarization → synaptic dysfunction	[54,55]
AIM2	Microglia; some astrocytes	Cytosolic dsDNA from damaged or stressed neurons/mitochondria accumulates in aging brain → AIM2 binds DNA → ASC recruitment	IL-1β, IL-18 secretion → amplifies Aβ deposition and tau hyperphosphorylation → worsens memory deficits	[56]
NLRC4	Microglia	ApoD induces NLRC4 inflammasome activation	ApoD-induced NLRC4 inflammasome activation in microglia → pro-inflammatory phenotype → impaired neural stem cell self-renewal and neuron apoptosis	[57]
